# *Burkholderia pseudomallei* transcriptional adaptation in macrophages

**DOI:** 10.1186/1471-2164-13-328

**Published:** 2012-07-23

**Authors:** Sylvia Chieng, Laura Carreto, Sheila Nathan

**Affiliations:** 1 School of Biosciences and Biotechnology, Faculty of Science and Technology, Universiti Kebangsaan Malaysia, Bangi, 43600, Malaysia; 2 CESAM and Departamento de Biologia, Universidade de Aveiro, Campus de Santiago, Aveiro, 3810-193, Portugal

**Keywords:** Burkholderia pseudomallei, Macrophage, Transcriptome analysis

## Abstract

**Background:**

*Burkholderia pseudomallei* is a facultative intracellular pathogen of phagocytic and non-phagocytic cells. How the bacterium interacts with host macrophage cells is still not well understood and is critical to appreciate the strategies used by this bacterium to survive and how intracellular survival leads to disease manifestation.

**Results:**

Here we report the expression profile of intracellular *B. pseudomallei* following infection of human macrophage-like U937 cells. During intracellular growth over the 6 h infection period, approximately 22 % of the *B. pseudomallei* genome showed significant transcriptional adaptation. *B. pseudomallei* adapted rapidly to the intracellular environment by down-regulating numerous genes involved in metabolism, cell envelope, motility, replication, amino acid and ion transport system and regulatory function pathways. Reduced expression in catabolic and housekeeping genes suggested lower energy requirement and growth arrest during macrophage infection, while expression of genes encoding anaerobic metabolism functions were up regulated. However, whilst the type VI secretion system was up regulated, expression of many known virulence factors was not significantly modulated over the 6hours of infection.

**Conclusions:**

The transcriptome profile described here provides the first comprehensive view of how *B. pseudomallei* survives within host cells and will help identify potential virulence factors and proteins that are important for the survival and growth of *B. pseudomallei* within human cells.

## Background

*Burkholderia pseudomallei* causes melioidosis, a disease with considerable human mortality and morbidity in the tropics [1]. Clinical presentation of melioidosis varies from rapidly fatal septicemia and acute pneumonia, chronic or localized abscess formation to skin and soft tissue infections that progress rapidly to systemic infection [2,3]. Treatment of melioidosis involves long periods of antibiotic administration which are vital to eliminate *B. pseudomallei* and to prevent relapse [4,5].

Several features of melioidosis suggest that *B. pseudomallei* is a facultative intracellular bacterium. These include long incubation periods of up to 62 years and the tendency to relapse [3,6]. *B. pseudomallei* has the ability to survive and proliferate within phagocytic and non-phagocytic cells [7,8], and free-living amoeba [9] for months or years. The exact mechanism of invasion is still unknown, but it requires the rearrangement of host actin cytoskeleton and the involvement of BopE, an effector protein of the type III secretion system (T3SS-3) [10]. After cellular uptake, *B. pseudomallei* escapes from endocytic vacuoles and once in the cytoplasm, induces host cell fusion and enters neighbouring cells by forming actin tails and membrane protrusions [11,12]. Vacuole escape and intracellular survival requires a functional T3SS-3, as mutants of T3SS-3 display delayed vacuolar escape, reduced actin formation and reduced capacity to survive intracellularly and spread to neighbouring cells [13,14]. It is possible that intracellular survival and intercellular spread may provide *B. pseudomallei* protection from host defences.

Recently, the type VI secretion cluster tss-5 (T6SS-1) was shown to contribute to intracellular growth of *B. pseudomallei*[15]. The expression of this secretion system is dependent on the sensor regulators BprC and VirA-VirG (VirAG). The type VI secretion system is also thought to play a major role during bacterial transition from the phagosome to the cytosol [15]. Moreover, T6SS is also implicated in other important bacterial pathogens as the key virulence factor and is involved in translocation of effector proteins into eukaryotic cells [16,17]. Using *in vivo* expression technology, Shalom et al. [18] found that the *B. pseudomallei tss-5* gene cluster was induced inside murine macrophages. Furthermore, Pilatz and colleagues demonstrated that a *B. pseudomallei tss-5* transposon mutant displayed reduced ability to form plaques on PtK2 epithelial cell monolayers, indicating the requirement of *tss-5* in cell-to-cell spread [19]. This mutant was also highly attenuated in mice with reduced bacterial load in the spleen, liver and lung at 48 h post-infection [19].

Previous studies have examined the detailed cellular responses of *B. pseudomallei* within eukaryotic cells. However, the overall picture of the mechanisms involved in adaptability to the intracellular lifestyle is still unclear. The availability of the complete genome sequence for *B. pseudomallei*[20] enabled us to design a whole genome DNA microarray to identify *B. pseudomallei* genes regulated during infection of macrophages. These data provide an insight into genes involved in survival and adaptation of the bacteria within macrophage cells as well as a new understanding of the biology of host-pathogen interaction during melioidosis.

## Results

### Infection model and bacterial RNA isolation

**Figure 1:**
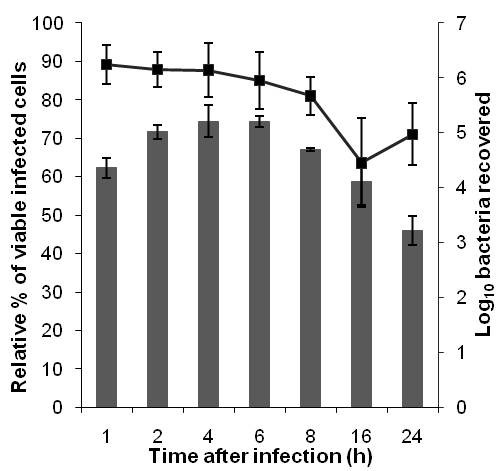
**B. pseudomallei intracellular growth and viability of infected U937.** Intracellular survival of *B. pseudomallei* was monitored over a 24 h period, along with U937 cell viability after *B. pseudomallei* infection. The relative amount of bacteria recovered (log_10_ scale) was plotted in grey bars while the relative % of viable infected cells was indicated as a solid line. Vertical lines represent the standard deviation (SD) obtained from three independent experiments conducted in triplicate (n = 9).

Human macrophage-like U937 cells were chosen as the infection model as it has previously been used extensively to study *B. pseudomallei* interaction with host cells [7,8]. In this study, bacterial replication within macrophages was observed with a maximum CFU obtained at 6 h post-infection. The calculated doubling time for intracellular *B. pseudomallei* is about 6.4 h, indicating a slower growth rate compared to the control bacteria in RPMI (doubling time of ~ 1.9 h). The number of viable bacteria decreased consistently after 6 h post-infection and a 2-log reduction in viable cells was noted with prolonged incubation of up to 24 h. A similar decline in bacterial numbers following prolonged incubation was previously reported in RAW264.7 cells [7,21]. Viability of infected U937 cells decreased over time and was drastically reduced by 6 h post-infection, suggesting a cytotoxic effect of *B. pseudomallei* on the host cells (Figure 1).

**Figure 2:**
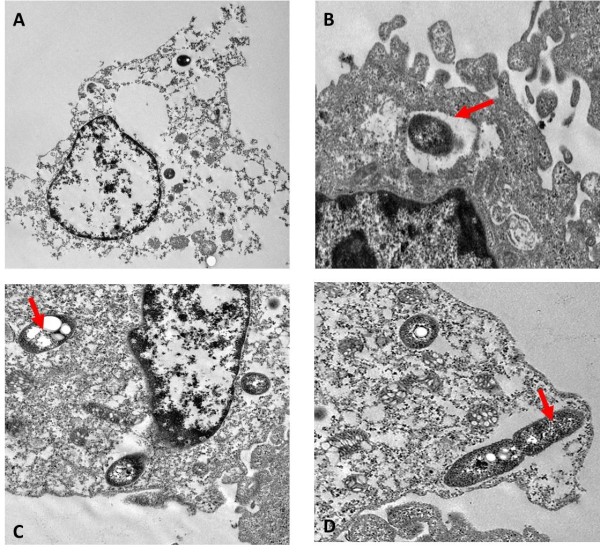
**Transmission electron micrograph of intracellular*B. pseudomallei*within U937 cells**. (A) *B. pseudomallei*-infected U937 cells demonstrating oncotic-like morphology and disruption of cell membrane. ×7000 (B) Internalised *B. pseudomallei* within membrane-bound phagosome (arrowed) were observed at early stage of intracellular infection. ×8000 (C) Loss of phagosome membrane was clearly visible and free bacteria were seen in the cell cytoplasm (arrowed). ×8000 (D) A dividing bacillus in a phagosome with near-intact membrane (arrowed). Some membrane dissolution can be seen close to the ends of the bacterial cell. ×8000.

An ultrastructural study of infected U937 cells showed that the nuclei, mitochondria and vacuoles were swollen whilst the nuclear material appeared diluted and the cell membrane totally disrupted (Figure 2A). All these changes indicate that infected U937 cells displayed a phenotype similar to oncotic cells as previously observed in other cells infected with *B. pseudomallei*[21]. Loss of phagosome membrane in *B. pseudomallei* infected macrophage cells was established as early as 15 min post-incubation similar to that previously reported [22]. At 2 h post-infection, loss of phagosome membrane is evident and bacilli were seen in the cytoplasm (Figure 2C). Moreover, replication of intracellular bacteria was seen in the cytoplasm of host cells (Figure 2D).

**Figure 3:**
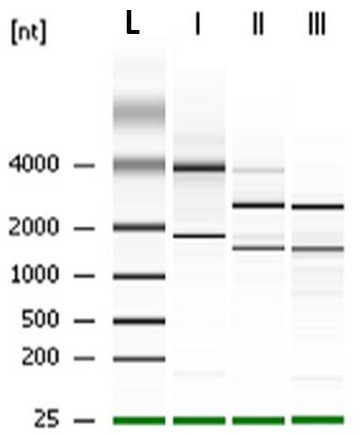
**Size chromatographic separation of RNAs**. Total RNA were extracted from (I) unifected U937 cells, (II) *B. pseudomallei* harvested from infected U937 cells and (III) *B. pseudomallei* grown *in vitro*. Lane L represents RNA ladder (kb).

An infection period of 1 to 6 h was selected to study changes in the expression profile as more than 85 % of the infected U937 cells were still viable during this period (Figure 1). This would ensure maximum recovery of intracellular *B. pseudomallei* from viable cells to obtain sufficient intact bacterial RNA. We adopted the differential lysis method with saponin to prevent bacterial cell lysis and easily recover bacterial RNA. This strategy enriches the bacterial mRNA several thousand-fold [23]. Treatment with saponin specifically lyses eukaryotic cells without affecting bacterial viability [24,25] and in our study, the use of 0.1 % to 4 % saponin was not detrimental to *B. pseudomallei* (data not shown). Differential centrifugation was combined with this method to efficiently remove host cellular debris. Based on the RNA integrity number (RIN) and the 16 S/23 S rRNA subunit ratio, the electrophoretic profiles of the total RNA extracted from intracellular bacteria showed no evidence of degradation and only minor host RNA contamination was detectable (Figure 3).

### The global gene expression profile

**Figure 4:**
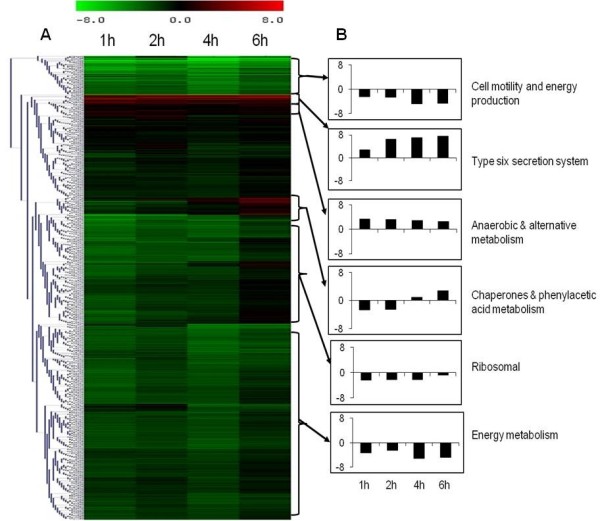
**Gene expression profile of*B. pseudomallei*during infection of U937 cells.** (A) The cluster diagrams show the expression profile of intracellular *B. pseudomallei* within U937 cells for 1 to 6 h relative to *in vitro* grown bacteria. Hierarchical clustering was performed with Euclidean correlation in TIGR-MeV. Expression values are determined from the SAM analysis with red representing up-regulation (ratio of + 8.0) and green representing down-regulation (ratio of - 8.0) on a log_2_ scale. (B) Average relative levels of expression of representative functional groups. The vertical axis shows the fold change in expression on a log_2_ scale. Data are the means from three biological replicates.

**Figure 5:**
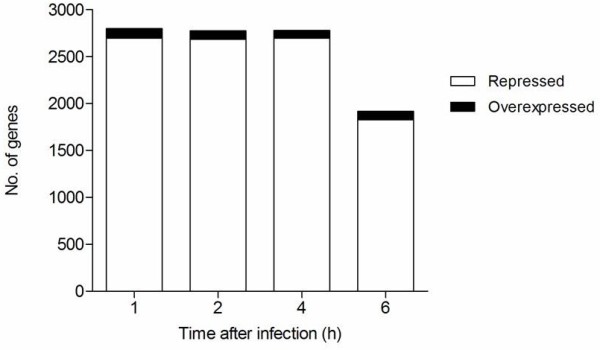
**Time-dependent transcriptional adaptation of*B. pseudomallei*in U937 cells.** The graph shows the total number of *B. pseudomallei* genes which are significantly differentially expressed (2-fold up or down, *p* < 0.01) at each time point.

**Figure 6:**
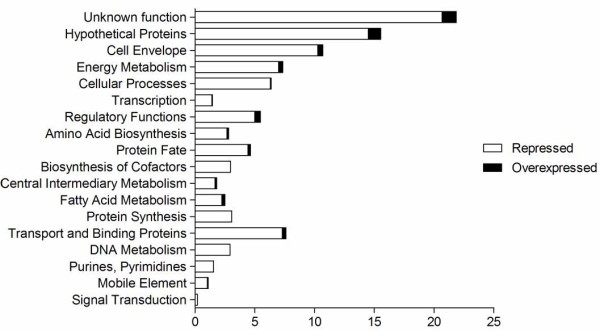
**Functional classification of intracellular*B. pseudomallei*regulated genes.** Bars indicate percentages of genes in each group that were significantly regulated at any time point. Genes were divided into functional categories based on Comprehensive Microbial Resources (CMR) annotations.

Temporal gene expression profiles obtained from intracellular *B. pseudomallei* were compared with the transcriptome of control bacteria grown in cell culture medium. Statistical analysis (*p* < 0.01) combined with a 2-fold variation cut off indicated that 2,797 genes were differentially expressed at 1 h, 2,755 at 2 h, 2,776 at 4 h and 1,918 genes at 6 h. Hierarchical clustering of gene expression levels revealed similar patterns of gene regulation over the period of infection (Figure 4A). The majority of the genes with altered expression were down-regulated throughout the infection period compared to bacteria grown in control medium. Expression levels of these genes continued to decrease up to 4 h before increasing to levels lower or similar to control bacteria at 6 h. It appears that the adaptation of *B. pseudomallei* within U937 cells is rapid and most of the changes occurred as early as 1 h post-infection (Figure 5). With time, the number of significantly deregulated genes gradually decreased, suggesting that *B. pseudomallei* had become adapted to the intracellular environment. Functional classification of intracellularly modulated bacterial genes at each time point showed that most of these genes encoded core functions such as metabolism, cell envelope, regulatory functions, transport and binding. Many genes encoding proteins with unknown function or hypothetical proteins were also modulated during infection (Figure 6).

Of the 5,721 probes represented on our microarrays, 1,284 transcripts representing 22.4 % of the *B. pseudomallei* ORFeome, were differentially expressed during the infection time course. Of these, 25 genes demonstrated increased expression (Table 1) while 1,259 genes were down-regulated ( Additional file 1). We performed gene function enrichment analysis on these genes to further categorize them into biological functions. Based on the statistical analysis of KEGG biochemical pathways, the number of genes involved in cellular functions such as biosynthesis of flagella and capsule, energy metabolism and regulatory systems was significantly higher during intracellular infection (Table 2).

**Table 1:** **Twenty-five common up-regulated genes of*B. pseudomallei*during intracellular growth in host macrophages relative to*in vitro*growth**

**Gene**	**Description**	**Fold Change (*in vivo*/*in vitro*) at the indicated time (h)**			
		1	2	4	6
BPSL0184	Putative rod shape-determining protein	23.83	15.31	12.69	12.06
BPSL0842	Benzoylformate decarboxylase	70.27	31.78	27.64	20.64
BPSL0886	Hypothetical protein	12.29	8.36	6.56	5.20
BPSL1067	Hypothetical protein	8.39	5.15	6.75	6.27
BPSL1771	Cobalamin biosynthesis protein CbiG	25.36	16.66	15.21	15.76
BPSL1817	Putative lipoprotein	30.07	14.11	12.67	11.33
BPSL1902	Hypothetical protein	37.91	18.81	12.46	8.39
BPSL2759	Putative short-chain dehydrogenase	16.54	8.31	5.93	5.61
BPSL2945	Allantoicase	27.90	9.57	8.83	7.41
BPSL3354	Putative cytochrome	18.66	11.70	8.64	7.62
BPSS0140	Sugar ABC transport system, lipoprotein	7.80	16.62	13.56	13.94
BPSS0142 *	Sugar ABC transport system, ATP-binding protein	4.62	10.04	11.34	18.79
BPSS0143	ROK family transcriptional regulator	4.78	9.97	11.80	18.64
BPSS0404	Methylamine utilization protein	49.46	36.31	30.14	23.42
BPSS0433	Hypothetical protein	11.87	5.84	7.70	10.59
BPSS0529	Lipoprotein	26.49	19.04	14.10	10.68
BPSS1279	Threonine dehydratase	17.47	11.84	10.80	11.03
BPSS1498 *	*tssD*, type VI secretion system Hcp protein	7.38	93.61	130.11	181.92
BPSS1499	*tssE*, type VI secretion system lysozyme	4.72	58.87	99.06	160.21
BPSS1505	*tagB*, type VI secretion system hypothetical protein	6.49	14.01	18.40	27.97
BPSS1508	*tssJ*, type VI secretion system lipoprotein	5.04	6.27	8.17	9.88
BPSS1728 *	Secretion/activator protein	3.00	23.60	29.33	24.54
BPSS1892	*catA*, catechol 1,2-dioxygenase	7.76	19.46	16.64	16.53
BPSS1893	*catC*, muconolactone delta-isomerase	10.57	9.30	7.33	6.30
BPSS2276	LysR family regulatory protein	59.21	41.83	26.33	19.45

Note: * Genes selected for real-time qPCR analysis.

**Table 2:** **Gene function enrichment analysis of*B. pseudomallei*common up-regulated and down-regulated genes throughout growth within host macrophages**

**Functional class or pathway**	**No. of genes regulated**	**No. of genes in genome**	**Significance (*p-*value)**
Up-regulated genes			
Benzoate degradation via hydroxylation	3	29	3.33 × 10^-2^
Down-regulated genes			
Amino sugar and nucleotide sugar metabolism	22	39	7.98 × 10^-10^
Bacterial chemotaxis	23	46	2.65 × 10^-9^
Lipopolysaccharide biosynthesis	13	24	2.36 × 10^-5^
Peptidoglycan biosynthesis	11	24	7.93 × 10^-4^
Flagella assembly	11	38	2.72 × 10^-2^
Alanine, aspartate and glutamate metabolism	15	32	2.41 × 10^-5^
Fatty acid biosynthesis	9	21	6.33 × 10^-3^
Two-component system	30	104	3.68 × 10^-6^
Glycolysis/gluconeogenesis	11	36	1.81 × 10^-2^
Oxidative phosphorylation	15	60	1.55 × 10^-2^

**Figure 7:**
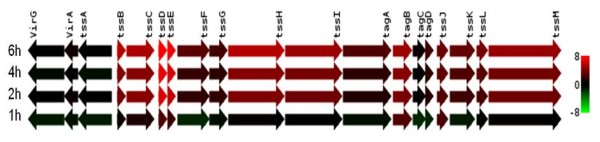
**Real-time quantitative PCR validation of microarray data.** Relative expression ratio by real-time qPCR (grey bars) or by microarray experiments (white bars) at 6 h post-infection. The horizontal axis represents fold change in log_2_ scale. Data are mean ± SD of triplicate measurements from three independent experiments.

As an independent measure of differential gene expression, we examined the relative expression of six up- or down-regulated genes selected from different functional categories by real-time qPCR (Table 1 and Additional file 1) on the same samples as those used for microarray analysis. Changes in expression were verified by real-time qPCR with a correlation of >0.95 to the microarray data (Figure 7). The strong correlation observed verified the efficiency and robustness of the designed microarray for high throughput screening of the *B. pseudomallei* transcriptome.

Due to the large number of significantly differentiated genes modulated during the infection, only data related to genes that have some functional information are shown and discussed below. The identified genes are discussed according to functional categories.

### Intracellular metabolism and ion transport

In host-pathogen interactions, sufficient nutrition levels are necessary for the successful survival of the pathogen. Once intracellular, the bacteria must make metabolic adjustments to adapt to changes in nutrient availability. We noted a robust shutdown in the expression of *B. pseudomallei* genes involved in metabolism. Genes involved in glycolysis and oxidative phosphorylation were consistently down-regulated in intracellular *B. pseudomallei*. In addition, most genes involved in energy metabolism such as ATP synthase and NADH dehydrogenase were down-regulated (Figure 4B). Collectively, these data suggest that intracellular *B. pseudomallei* have lower energy requirements and limit their energy production during the initial stage of infection. Several anaerobic metabolism genes were induced in intracellular *B. pseudomallei*. Anaerobic metabolism pathway genes such as BPSS1279 (threonine dehydratase), BPSL1771 (cobalamin biosynthesis protein CbiG) and BPSS0842 (benzoylformate decarboxylase) were up-regulated throughout the infection period. Nevertheless, none of the components of the anaerobic respiratory chain showed significant changes in expression except for BPSL2311 (putative respiratory nitrate reductase delta chain) and BPSL2312 (putative respiratory nitrate reductase gamma chain) that were induced at the early stage of infection.

Other induced genes were *catAC* genes, which are involved in benzoate degradation, indicating that intracellular *B. pseudomallei* utilized aromatic compounds as a source of carbon. Increased expression of phenylacetic acid (PA) pathway genes at the later stage of infection (4 h and 6 h post-infection) was also observed (Figure 4B). The major nitrogen source in the intracellular compartment is most likely methylamine and purine as suggested by the increased expression of methylamine utilization protein (BPSS0404) and allantoicase (BPSL2945). Allantoicase is involved in purine metabolism and provides a secondary nitrogen source under nitrogen limiting conditions [26].

### Expression of virulence and virulence-associated factors

We observed the repression of genes encoding proteins that are well characterised as *B. pseudomallei* virulence factors. These include the main capsular polysaccharide biosynthesis (BPSL2787-BPSL2810) genes, two potential surface polysaccharide biosynthesis gene clusters (BPSS0417-BPSS0429 and BPSS1825-BPSS1834), majority of genes in the lipopolysaccharide (LPS) biosynthesis cluster and genes encoding for flagella assembly and chemotaxis. We also noted the repression of *bspR*, a regulator recently shown to control the expression of TT3SS-3 genes [27]. The inhibition of this regulator leads to the reduced expression of T3SS-3 controlled genes in intracellular *B. pseudomallei*.

**Figure 8:**
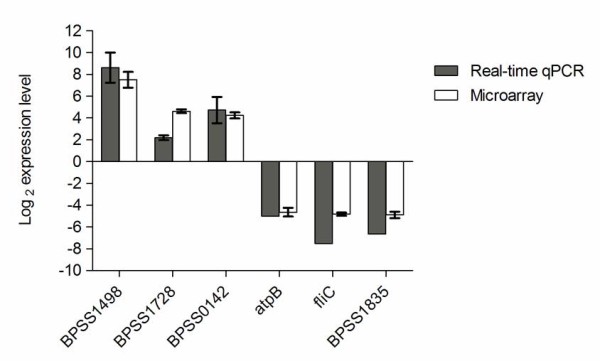
**Regulation of the type VI secretion system tss-5 cluster.** The heat-map shows the expression ratio of each gene in the T6SS tss-5 cluster during intracellular growth in host macrophages relative to *in vitro* growth. Expression values are determined from the SAM analysis with red representing up-regulation (ratio of +8.0) and green representing down-regulation (ratio of -8.0) on a log_2_ scale.

One of the six clusters of the type VI secretion system, the tss-5 cluster (BPSS1493-BPSS1511), was up-regulated up to 182-fold during intracellular infection (Figure 8). This observation is consistent with previous reports on the induction of three genes in this cluster, *tssH-5tssI-5* and *tssM-5*, upon invasion of macrophages [18]. We also observed the induction of genes flanking the tss-5 cluster, *bimA* (*Burkholderia*intracellular motility A)(BPSS1492) and BPSS1512 at 2 to 6 h post-infection. Moreover, the hemolysin activator-like protein precursor, *fhaC* (BPSS1728) gene was significantly up-regulated during intracellular infection. Consistently, the large filamentous hemagglutinin precursor, *fhaB* (BPSS1727) gene, a potential virulence factor of *B. pseudomallei*[20], was induced between 2 to 6 h post-infection.

### Stress responses genes

The general stress-responsive alternative sigma factor *rpoS* transcribes genes involved in bacterial survival under conditions of environmental stress. In *B. pseudomalleirpoS* is a regulator of carbon starvation and oxidative stress [28].We observed down-regulation of *rpoS* throughout intracellular growth. Furthermore, the expression of most of the genes encoding sigma factors was repressed in the intracellular bacteria. Under oxidative stress, *rpoS* regulates *oxyR* and *katGdpsA* operons [29]. As expected, we observed down regulation of *oxyR* and *katGdpsA* expression at 1 and 2 h post-infection. The expression of other genes in the *oxy R* regulon was either repressed or did not change significantly relative to control cells. Moreover, class I stress response genes including *dnaJdnaKhrcA* and *groEL* were not significantly regulated, except for *groES*, which was induced at 6 h post-infection.

### DNA topology and growth arrest within macrophages

Modification of DNA topology plays a major role in assisting DNA replication and protein synthesis. Prokaryotic DNA is usually maintained in a negatively supercoiled form by topoisomerases and the level of supercoiling can be affected by several environmental parameters [30]. The ability of DNA gyrase to generate negative supercoils in DNA is inhibited when ATP levels are reduced [31]. In this study, DNA topoisomerase IV subunit A (*parC*), DNA topoisomerase IV subunit B (*parB*) and DNA gyrase subunit A (*gray*) genes were down-regulated. The repression of gyrase suggests a relaxation in bacterial DNA supercoiling during *B. pseudomallei* intracellular growth in U937. Additionally, the nucleotide-associated regulator protein Fis was also negatively regulated. Fis expression depends on superhelical density whereby maximum density is observed at high levels of negative supercoiling [32].

We found that bacterial genes involved in cell division (*ftsABH*) were down-regulated indicating reduced cell division activity. The repression of *minD* and *minE* genes, which mediate spatial regulation of cytokinesis in bacteria [33], and chromosome partitioning genes, *parA* and *parB* was also observed. Furthermore, replicative DNA helicase *dnaB*, DNA polymerase III subunit alpha *dnaE* and DNA polymerase III subunit chi genes were also down-regulated. Consistent with the down regulation of catabolic and cell replication genes, the expression of RNA polymerase (*rpoABCZ*) and ribosomal subunit genes (S1, S10, S21, L11 and L28) was also reduced throughout the infection period. Collectively, these support the view that cell division and replication processes are interrupted during infection, leading to slower growth kinetics of intracellular *B. pseudomallei*.

## Discussion

Numerous studies have been conducted on the cellular interaction between *B. pseudomallei* and eukaryotic cells, especially the ability of this pathogen to survive within host cells. In this study, we investigated the global *B. pseudomallei* transcriptome profile during early stage infection of human monocyte-like U937 cells. Initially, we demonstrated that replication kinetics of intracellular *B. pseudomallei* within U937 cells is similar to data from a number of previous studies [8,34]. The bacteria were able to survive in macrophage cells, albeit, at a slower growth rate compared to bacteria grown in RPMI. Reduced bacterial growth kinetics during infection was also reported in a hamster model of melioidosis [35]. Furthermore, this reduced bacterial replication kinetics in the intracellular environment in comparison with *in vitro* growth is not limited to *B. pseudomallei*[36-38]. *B. pseudomallei* infection was also cytotoxic to macrophage cells with about 20 % cell mortality at 24 h post infection which, to some extent, explains the reduced number of recovered bacteria with prolonged incubation. Electron micrographs of infected U937 cells revealed disruption of internal structures and formation of oncotic cells. The cytotoxic and cytopathy effects of *B. pseudomallei* infection in our study are in agreement with Sun et al. [21], who reported high cytotoxicity (40-70 %) and oncotic death in infected THP-1 cells. We also observed the loss of phagosome membrane in infected macrophages as early as 2 h post-infection resulting in the presence of free replicating bacilli in the cytoplasm at later time points.

The ability to survive within and lyse macrophage cells most probably contributes to the spread of this bacterium and disease progression. We attempted to identify mechanisms that underlie the adaptive ability of *B. pseudomallei* during infection by comparing intracellular bacteria transcripts to that of *in vitro* grown bacteria. During infection, we observed that intracellular *B. pseudomallei* demonstrated a lower energy requirement and production by shutting down metabolic activity. The reduced expression of catabolic and house-keeping genes is consistent with the reduced growth rate observed for intracellular *B. pseudomallei*. The induction of anaerobic metabolism pathway genes is suggestive of limited oxygen concentrations in the cytosol although expression of genes of the anaerobic respiratory chain was not significantly altered. The importance of this finding requires further investigation.

*B. pseudomallei* utilizes alternative metabolites during infection [35]. Our study suggests that *B. pseudomallei* utilize aromatic carbon compounds such as benzoate and PA as carbon sources for intracellular survival. PA can inhibit the inducible nitric oxide synthase (iNOS) and LPS-induced expression of cytokines in rat primary astrocytes, microglia and macrophages [39]. Moreover, PA can repress DNA binding and transcriptional activities of NFκB, an important upstream modulator for cytokine and iNOS expression in macrophages [40]. The up-regulation of PA catabolic pathway genes in intracellular bacteria might partly explain why macrophages infected with *B. pseudomallei* failed to activate the production of iNOS [41]. The putative *B. cenocepacia* ring PA-coenzyme A hydroxylation system was found to be essential for full pathogenicity in infected *Caenorhabditis elegans*[42].

There are three T3SSs present in *B. pseudomallei* and only T3SS-3 is involved in virulence [43]. *B. pseudomallei* T3SS-3 shows high homology to the Inv/Mxi-Spa type III secretion systems of *Salmonella* and *Shigella*[13]. As T3SS-3 is known to be essential in facilitating invasion and early phagosomal escape into the cytosol [13,14], the down-regulation observed in our study is consistent with this function as it would be conceivable that T3SS-3 is important at the beginning of intracellular life during invasion, but not important at the later stages of intracellular infection. Similarly, the role of T6SS-1 is reported to be crucial during the transition from phagosome to cytosol [15]. Thus, it is possible that the induction of T6SS-1 in this study plays a major function in ensuring pathogen survival and replication in the cytosol. In *B. mallei,* a close related species of *B. pseudomallei,* an effector protein of T6SS (BMAA0742) was recognized by glanders antiserum, indicating production of this protein *in vivo* during infection [44]. In this study, high induction of *tssD-5* (BPSS1498), an effector Hcp1 protein of T6SS was observed throughout the infection period. A recent study on *B. pseudomallei* T6SS Hcp proteins has shown that *tssD-5* deletion mutant was attenuated in a hamster infection model, exhibited growth defects and was only weakly cytotoxic to RAW264.7 macrophages [45]. Additionally, human melioidosis serum samples were found to react with Hcp1 protein, consistent with our observation (data not shown). These results suggest that Hcp1 is produced by *B. pseudomallei in vivo* during infection, is immunogenic and vital for intracellular survival of *B. pseudomallei*.

The genes *bimAfhaB* and *fhaC* are known proteins that mediate actin tail formation involved in cell-to-cell spread. In this study, expression of these three genes is up-regulated. The over expression of BimA has previously been shown in intracellular *B. pseudomallei* and mutation of *bimA* abolished actin-based motility of this pathogen in J774.2 cell [46]. In *Bordetella pertussis*, the FhaC dependent filamentous hemagglutinin (FHA) facilitates attachment to the host cell during infection [47] and initiates killing of macrophages to avoid the host cell mediated immune response whilst infection is being established [48]. Through a genome-wide function screen of *B. pseudomallei* strain K96243, both *fhaB* and *fhaC* were found to form part of the anti-macrophage loci and contribute to the formation of dramatic actin projections extending towards neighbouring cells [49].

Interestingly, in our study, well-characterized virulence genes of *B. pseudomallei* such as capsular polysaccharide, LPS and flagella were either repressed or not significantly expressed. Similar profiles were noted in a hamster model, wherein most of the genes encoding for capsule, LPS and flagella biosynthesis were either down-regulated or not significantly changed in expression [35]. This could be due to the equal expression of these genes under *in vivo* and *in vitro* conditions concomitant with previous evidence that capsule, LPS, flagellum and other virulent determinants are constitutively produced *in vitro*[35]. A recent report of a similar study on macrophages infected by *B. cenocepacia* demonstrated that this intracellular pathogen’s ability to adapt and respond to the intracellular milieu was not dependent on the expression of any specific virulence-associated factors [50]. Hence, this ability to adapt to various niches appears to be conserved amongst the soil-derived Burkholderia spp. clinical pathogens. Down regulation of flagella genes and subsequently the absence of flagellin, may also reflect the bacteria’s attempt to limit immune recognition especially by toll-like receptors by modulating surface structures. In Gram negative bacteria, flagellin is recognized by TLR5 which triggers the secretion of various cytokines and chemokines, leading to an inflammatory response [51]. By dampening the activation of TLR5, the innate immune response towards *B. pseudomallei* is reduced and bacterial clearance is prevented. Consistent with our observations, *B. pseudomallei* isolates failed to significantly increase TLR5 expression in both cell culture [52] and BALB/c mice infection models [53]. Nanagara et al. [54] also suggested that the suppression of *B. pseudomallei* surface antigens in naturally infected human synovial tissues aids in bacteria survival against host immune responses and antibiotic treatment.

Intracellular bacteria are constantly exposed to a range of stresses such as oxidative burst and formation of oxygen radicals by the host cellular defence mechanisms. Several *in vitro* studies designed to mimic the host environment have identified the expression of the *dspA*[55] and *ahpC-katG*[56] genes as necessary for survival and growth under oxidative stress. Surprisingly, in our study, expression of the majority of oxidative stress related genes was repressed or not significantly altered. This observation suggests the ability of *B. pseudomallei* to rapidly evade oxidative stress by escaping from the phagosome [22]. This enables the bacteria to enter the cytoplasm, a more favourable environment for survival and growth where nutrients are freely available and microbicides do not operate [7].

## Conclusions

This is the first report of a complete transcriptome profile of intracellular *B. pseudomallei* within macrophages. We have determined that *B. pseudomallei* adapts rapidly to the intracellular environment through the regulation of bacteria metabolism and growth rate and the possibility of host cell immune response avoidance through shutdown of known virulence factors. Proteins encoded by genes induced during infection including genes encoding the T6SS cluster are potential diagnostic candidates or targets for anti-microbial development.

## Methods

### Bacterial strain and growth conditions

*B. pseudomallei* D286, a clinical isolate previously described by Lee [57], was grown on Ashdown agar for 48 h or in Luria-Bertani (LB) broth overnight at 37 °C. Prior to infection, overnight cultures were diluted to 1:50 in LB broth and grown to mid-logarithmic phase (OD_600_ = 0.4-0.6) at 37 °C, 250 rpm for 3 h.

### Cell culture and infection model

Human monocyte-like U937 cells (CRL-1593.2) were maintained in RPMI1640 medium (Gibco) supplemented with 10 % fetal bovine serum (Hyclone), 2 mM L-glutamine, 10 mM HEPES and 1 mM sodium pyruvate (Invitrogen). For infection assays, 2 × 10^5^ cells/well were seeded in 12-well cell culture plates, while for RNA extraction, approximately 3 × 10^7^ cells/flask were seeded in T175 flasks. U937 cells were supplemented with 10 ng/ml phorbol myristate acetate (PMA) (Sigma) to induce macrophage differentiation 48 h prior to infection [58]. Induced U937 cells were washed once with Hanks’ Balance Salt Solution (Gibco) to remove traces of PMA before addition of bacteria at a multiplicity of infection of 10. After 2 h at 37 °C, extracellular bacteria were removed by extensive washing with PBS. Fresh media containing 250 μg/ml kanamycin was added to each plate or flask and infected cells were incubated at 37 °C until lysed.

### Intracellular survival and eukaryotic cell viability determination

Intracellular survival of *B. pseudomallei* in U937 was estimated as previously described with some modifications [8]. At different time points following the initial 2 h infection with *B. pseudomallei*, infected macrophage monolayers were washed three times with PBS and intracellular *B. pseudomallei* were harvested by adding 500 μl of 1 % saponin in PBS to each well. After 5 min incubation at 37 °C, cell lysates were collected and serially diluted 10-fold in PBS and aliquots were plated onto Ashdown agar to assess viable bacterial counts.

To determine the overall viability of macrophage cells following bacterial infection, trypan blue exclusion was used. At selected time points after infection with bacteria, the monolayers were washed three times with PBS and gently scrapped off from the wells. Trypan blue solution was added to cell suspensions and stained infected cells were visualized under an inverted microscope. Assays were performed in triplicate and repeated at least three times. The number of intact viable cells was expressed as a percentage relative to viable uninfected cells.

### Transmission electron microscopy

Transmission electron microscopy for infected U937 cells was performed as previously described [22] with some modifications. Briefly, cells were fixed for 24 h in 4 % glutaraldehyde at 4 °C and washed three times in PBS. After a secondary fix for 2 h in 1 % osmium tetroxide at 4 °C, the specimens were washed three times in PBS. The specimens were then dehydrated in a graded series of acetone/water containing 30 % to 100 % acetone. The specimens were infiltrated with acetone-resin mixture, embedded into resin hard mix and left to polymerize at 60 °C for 24 to 48 h. Ultrathin sections were stained with lead citrate and viewed on a Hitachi H-7100 transmission electron microscope.

### RNA extraction

At each time point (1, 2, 4 and 6 h post-infection), macrophage monolayers were washed and infected macrophages from three T175 flasks were combined and lysed in 1 % saponin in PBS (sterile and filtered) for 5 min at 37 °C [25]. Lysates were collected and subjected to differential centrifugation; first at 800 × *g* for 5 min to sediment eukaryotic cells and cellular debris, and secondly at 8,000 × *g* for 10 min to pellet bacterial cells. Pellets were immediately snap-frozen in liquid nitrogen and kept at -80 °C until extraction. Bacterial RNA was prepared using the Qiagen’s RNeasy Mini Kit and on-column DNase I digestion was performed. Total RNA obtained was further purified by ethanol/ammonium acetate precipitation. Control RNA from *in vitro* grown bacteria was obtained by diluting overnight cultures and growing stationary at 37 °C to mid-logarithmic phase in complete RPMI1640 medium under 5 % CO_2_. These conditions mimicked those used for the cell infection experiments. Control bacteria were treated similarly and RNA was isolated as described above. Control U937 RNA was isolated using Trizol and purified with the RNeasy Mini Kit. The concentration, quality and integrity of all RNA isolated were analysed using the Nanodrop® ND-1000 and Agilent 2100 Bioanalyser.

A preliminary control experiment demonstrated that incubation in 1 % saponin for 5 min at 37 °C did not affect the viability of *B. pseudomallei* (data not shown). Additionally, when comparing the transcriptional profile of *B. pseudomallei* before and after treatment with saponin and differential centrifugation, we found that these treatments caused no significant changes in bacterial gene expression (data not shown), consistent with studies on other bacterial pathogens [24,59].

### Construction of *B. pseudomallei* DNA microarrays

Microarray containing probes to the annotated ORFs of the *B. pseudomallei* reference strain K96243 was designed using Agilent’s eArray 5.0 web-based tool and synthesized using Agilent’s 60-mer Sure Print technology. Probes were filtered using a perfect match filter to eliminate probes with identical sequences and a similarity score filter to discard probes with significant similarity to other parts of the target genome. Quality control of the probes was also done based on their base composition (BC) score. BC score is a numerical value that defines the quality of the probe based upon its base composition and distribution, with BC_1 being the best and BC_Poor the worst. Filtered probes with higher BC scores, namely BC_1 and BC_2, were included in the array, while probes with lower BC scores, such as BC_3 and BC_4, were excluded. A total of 5,721 probe sequences passed the filtering and quality control criteria and were replicated and randomly distributed in a microarray of 15,000 probes to fit Agilent 8 × 15 K microarray format. In summary, the *B. pseudomallei* 8 × 15 K microarrays (GEO reference GPL13233) allowed for the analysis of 5,721 non-cross-hybridizing ORFs of *B. pseudomallei* K96243.

### Sample labelling and hybridization

Prior to labelling, bacterial RNA was polyadenylated and reverse transcribed. Polyadenylation of bacterial RNA was based on the PAP method using A-plus^TM^Poly(A) Polymerase Tailing kit (Epicentre). Poly-a tailing was carried out to provide a priming site for the synthesis of first strand cDNA. Polyadenylation was terminated by ethanol/ammonium acetate precipitation. cDNA synthesis, labelling and hybridization were done according to the Agilent one-colour microarray protocols (available at http://www.chem.agilent.com/Library/usermanuals/Public/G4140-90040_GeneExpression_One-color_v6.5.pdf). Washes were also conducted according to standard Agilent protocols. An additional wash with acetonitrile was conducted to completely dry the arrays. Arrays were scanned with the Agilent Technologies Scanner model G2505B. Spot intensities and other quality control features were extracted with Agilent’s Feature Extraction Software version 9.5.3.1. In this study, 3 independent hybridizations using RNA samples isolated from 3 separate assays (biological replicates) were performed for each incubation time point and control bacteria.

### Data analysis

Microarray quality was assessed through the use of Agilent’s control features and only arrays that passed the recommended criteria were included in the analysis. Processed signals obtained from Feature Extraction were used as signal intensities for analysis. The data was filtered using a signal to noise ratio criteria and only features for which the background-subtracted signal was 2.6 times above the background standard deviation for that feature in at least 13 of the 15 arrays, were retained. A total of 5,391 genes passed the filtering process. Arrays were median normalized with BRB-ArrayTools (http://linus.nci.nih.gov) to adjust the scale of intensities across samples and arrays. Filtered and normalized data were subjected to Significance Analysis of Microarray (SAM) analysis in which a two class unpaired analysis was performed and genes with a false discovery rate (FDR) < 0.01 and fold change ≥ 2 were defined as significantly differentially expressed. Hierarchical clustering analysis with Euclidean correlation was performed using TIGR-MeV software version 4.3.2 (http://www.tigr.org). Functional classifications were carried out based on Comprehensive Microbial Resources (CMR) annotations (http://www.cmr.jcvi.org). Gene function enrichment analysis was performed on the DAVID 6.7 database (http://david.abcc.ncifcrf.gov/home.jsp) [60] using a Fisher Exact test with Benjamini and Hochberg multiple testing correction (*p* < 0.05). All microarray results have been deposited in the GEO database (http://www.ncbi.nlm.nih.gov/geo/) with the GEO series accession number GSE27558.

### Real-time quantitative PCR

Total RNA was treated with DNase I (Invitrogen) to remove any traces of DNA and converted to cDNA using Superscript III (Invitrogen) with random hexamers, according to the manufacturer’s instructions. Real-time quantitative PCR was performed on 10 ng cDNA in a final volume of 20 μl in a SDS7500 with Power Sybr Green PCR Master Mix (Applied Biosystems). All experiments were conducted three times, which yielded 9 measurements per gene (representing 3 technical replicates of 3 biological replicates) and the relative expression ratios were calculated using REST-MCS [61]. The gene coding for cytochrome d ubiquinol oxidase subunit II, *cydB*, was used as the reference gene for normalization. *CydB* was chosen as the reference gene because it did not show any significant changes in expression in the microarray experiment. Primers are described in Table 3. The correlation between expression ratios obtained from the microarray and real-time qPCR was evaluated with Pearson correlation (Microsoft Office Excel).

**Table 3:** List of oligonucleotides used in real-time qPCR experiments

**Gene**	**Primer name**	**Nucleotide sequence (5’- 3’)**
*cydB*	*cydB*_F *cydB*_R	GATCCGAAGAGCAGCC
		CAGCCCGTGTAGAGCAG
BPSS0142	BPSS0142_F	ACCGATAACCTGTTCCG
	BPSS0142_R	CGTAGATTTCCGCCATC
BPSS1498	BPSS1498_F	TCAAGGTCAAAGGAAAAAC
	BPSS1498_R	AAGGCGAGGATGTGGAT
BPSS1728	BPSS1728_F	AGAGCCGCCAAGATCAA
	BPSS1728_R	GCCGAGACCCGAGTTAT
*atpB*	*atpB*_F *atpB*_R	GTGGCTTTAACGATATGGC
		TGATGCGAGGTGGAGAA
*fliC*	*fliC*_F *fliC*_R	CAGACGAACTACAACGGC
		ATGCTTTGCGTGAGGTC
BPSS1835	BPSS1835_F BPSS1835_R	CGTGAAGAAAATCGTCG GAGTCGTAATGTCCCCAC

## Competing interests

The authors declare that they have no competing interests

## Authors’ contributions

SC contributed to the design of the study, performed all the experiments and data analysis and wrote the manuscript. LC contributed to the microarray interpretation and analysis and helped in performing real-time quantitative PCR. SN conceived and coordinated the study, contributed to the experimental design and wrote the manuscript. All authors read and approved the final manuscript.

## Supplementary Material

Additional file 1 List of 1259 common down-regulated genes of *B. pseudomallei* during intracellular growth in host macrophages relative to *in vitro* growth. Click here for file
